# Remdesivir postexposure prophylaxis limits measles-induced “immune amnesia” and measles antibody responses in macaques

**DOI:** 10.1172/jci.insight.190740

**Published:** 2025-04-22

**Authors:** Andy Kwan Pui Chan, Liting Liu, William R. Morgenlander, Manjusha Thakar, Nadine A. Peart Akindele, Jacqueline Brockhurst, Shristi Ghimire, Maggie L. Bartlett, Kelly A. Metcalf Pate, Victor C. Chu, Meghan S. Vermillion, Danielle P. Porter, Tomas Cihlar, Michael J. Mina, H. Benjamin Larman, Diane E. Griffin

**Affiliations:** 1 W. Harry Feinstone Department of Molecular Microbiology and Immunology, Johns Hopkins Bloomberg School of Public Health, Baltimore, Maryland, USA.; 2 Graduate Program in Immunology,; 3 Department of Pathology,; 4 Division of Pediatric Infectious Diseases, and; 5 Department of Molecular and Comparative Pathology, Johns Hopkins University School of Medicine, Baltimore, Maryland, USA.; 6 Gilead Sciences Inc., Foster City, California, USA.; 7 Immune Observatory, Boston, Maryland, USA.

**Keywords:** Immunology, Infectious disease, Virology, Adaptive immunity

## Abstract

Measles remains one of the most important causes of worldwide morbidity and mortality in children. Measles virus (MeV) replicates extensively in lymphoid tissue, and most deaths are due to other infectious diseases associated with MeV-induced loss of circulating antibodies to other pathogens. To determine whether remdesivir, a broad-spectrum direct-acting antiviral, affects MeV-induced loss of antibody to other pathogens, we expanded the VirScan technology to detect antibodies to both human and macaque pathogens. We measured the antibody reactivity to MeV and non-MeV viral peptides using plasma from MeV-infected macaques that received remdesivir either as postexposure prophylaxis (PEP) (d3–d14) or as late treatment (LT) (d11–d22) in comparison with macaques that were not treated. Remdesivir PEP, but not LT, limited the loss of antibody to non-MeV pathogens. Remdesivir PEP also limited the antibody response to MeV with a decrease in both the magnitude and breadth of the epitopes recognized. LT had little effect on the magnitude of the MeV-specific antibody response but affected the breadth of the response. Therefore, early, but not late, treatment of measles with the direct-acting antiviral remdesivir prevents the loss of antibody to other pathogens but decreases the response to MeV.

## Abstract 2

Given early after measles virus infection, the direct acting antiviral remdesivir mitigates the loss of antibody to other pathogens but decreases the response to measles virus.

## Introduction

Measles, a highly contagious systemic viral disease, remains one of the most important causes of worldwide morbidity and mortality in children (1, 2). Measles virus (MeV), the causative agent of measles, is an enveloped negative-sense RNA virus with a 16 kb nonsegmented genome that belongs to the genus *Morbillivirus* in the family *Paramyxoviridae*. The genome encodes 6 structural proteins and 2 nonstructural proteins. Two proteins are in the viral envelope: H, hemagglutinin, is the attachment protein; F, fusion, fuses the virus and cell membrane for entry; and M, matrix, links the surface glycoproteins with the ribonucleocapsid for assembly. The genome is surrounded by nucleocapsid (N) protein associated with phosphoprotein (P) and large polymerase (L) replication factors. The 2 nonstructural regulatory proteins V and C are encoded within the P gene (3). WT MeV is transmitted by aerosol or respiratory droplets (4) and spreads from the respiratory tract to local draining lymph nodes, where it infects immune cells expressing the signaling lymphocytic activation molecule 1 (SLAMF1/CD150) receptor (5). Infected peripheral blood mononuclear cells (PBMCs) spread virus systemically to multiple sites including lymphoid tissues of the thymus, spleen, gastrointestinal track, and epithelial and endothelial cells in nonlymphoid organs such as skin, conjunctivae, kidney, lung, and liver (6).

Measles has a profound effect on the immune system leading to immune suppression as well as to lifelong immunity to reinfection (7). Most measles-attributable deaths are caused by secondary infections (8), and survivors have an increased long-term need for antibiotic treatment and hospitalization compared with children who have not had measles (9–12). As a possible explanation for this increased susceptibility to infection, recent studies using VirScan, a phage-display immunoprecipitation and sequencing (PhIP-Seq) technology for high-throughput analysis of antibody binding (13), have documented a decreased amount and diversity of antibodies to other previously encountered pathogens, which may increase susceptibility to reinfection with these agents (14). This decrease is presumed to be due primarily to the loss of long-lived plasma cells (LLPCs) from the bone marrow caused directly by MeV-induced or immune-mediated cell death or caused indirectly by eviction from bone marrow niches as part of the immune response to infection (15).

*Macaca mulatta*, also known as rhesus macaque (RM), provides a well-characterized model system for MeV pathogenesis that closely resembles disease in humans (16). Previous studies have shown that postexposure prophylaxis (PEP) of macaques with i.v. remdesivir (RDV), a direct-acting broad spectrum antiviral, beginning 3 days after infection with WT MeV profoundly suppresses levels of viral RNA in blood during treatment but does not improve clinical disease (17). Replicating virus remains recoverable from PBMCs, and viral RNA levels rebound upon cessation of treatment. Development of MeV-specific antibodies is affected by treatment. In PEP-treated animals, titers of MeV-specific IgM are diminished, and MeV-specific IgG plateaued at a lower level with lower avidity than controls. However, a potential effect on antiviral antibody specificity has not been examined. In contrast, initiation of treatment on day 11 after infection at the onset of clinical disease had little effect on levels of viral RNA or antibody.

In the current studies, we have expanded the VirScan technology to include a panel of macaque, as well as human, pathogen peptides and assessed the effect of direct antiviral treatment on measles-induced loss of antibody diversity to other pathogens and on MeV-specific antibodies across longitudinal time points. PEP with RDV, but not later treatment, blunted the loss of antibody diversity, suggesting that early therapeutic intervention could decrease the morbidity and mortality from the measles-induced susceptibility to other infections.

## Results

### Expansion of the PhIP-Seq VirScan library to include macaque pathogens.

The original macaque studies of the changes in measles-induced antibody diversity were based on recognition of peptides from human pathogens cross-reactive with macaque pathogens (14). To increase the sensitivity and relevance for studies in macaques, we constructed a VirScan macaque pathogen library consisting of 5,682 nonhuman primate pathogen peptide sequences. Proteins from viruses that infect nonhuman primates were identified in the UniProt database, collapsed to 90% identity, and bioinformatically parsed into 56 amino acid peptide sequences with 28 amino acid overlaps between adjacent tiles using the pepsyn Python package (https://github.com/lasersonlab/pepsyn; commitID a596921).

### Longitudinal antibody reactivity to human and macaque pathogens.

Plasma samples were collected, and antibody reactivities to human and macaque pathogens were analyzed by VirScan from 14 previously studied RMs (17). These macaques have been intratracheally infected with WT MeV and received either RDV PEP beginning at day 3 (*n* = 5), RDV late treatment (LT) beginning at day 11 (*n* = 3), or vehicle (*n* = 6) i.v. for 12 days. Plasma samples were collected before infection and at approximately 2 (d14 or d15 [d14/d15]), 4 (d28), 12 (d84/d89) and 16–25 weeks (d112/d168/d176) after infection. In addition, plasma samples from uninfected, untreated macaques (*n* = 4) were similarly analyzed as controls.

**Figure 1:**
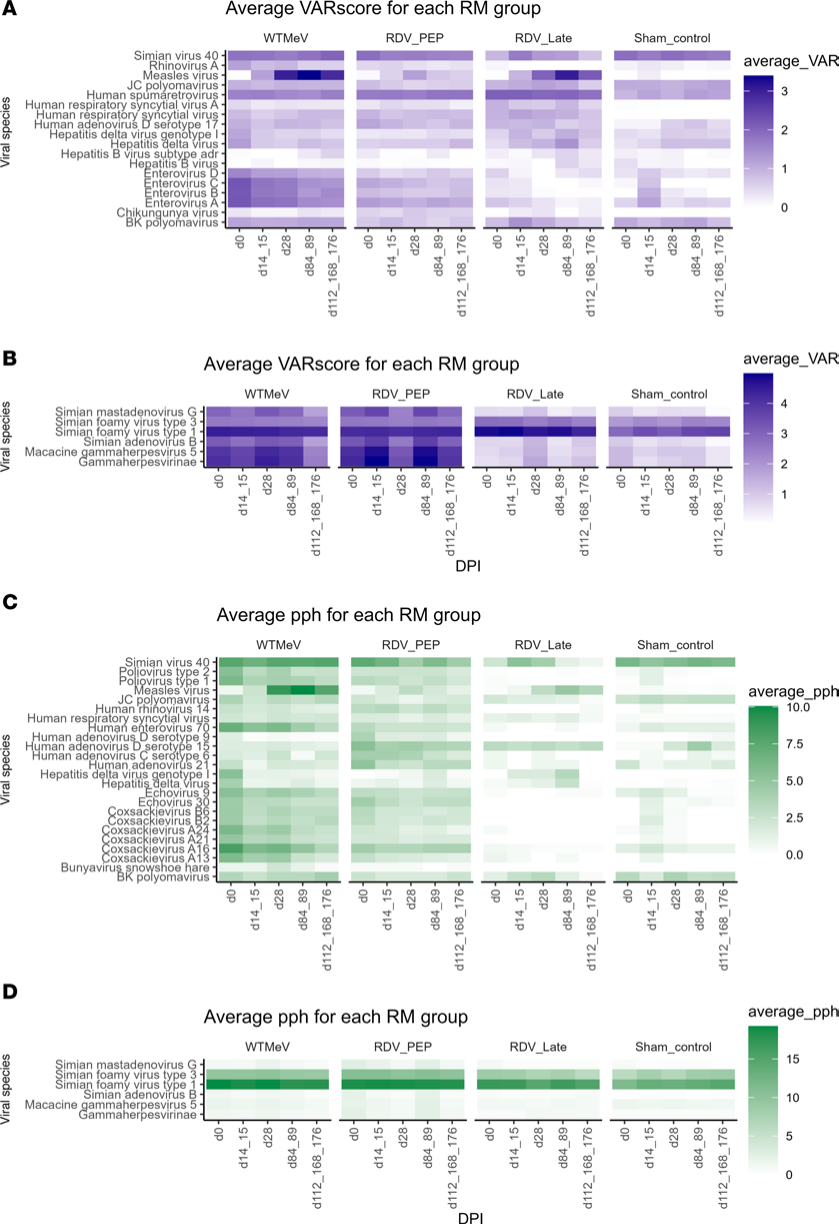
Effect of remdesivir administration to MeV-infected rhesus macaques on longitudinal average VARscore and percentage peptide hits (PPH) for human and macaque viral species. (**A**–**D**) Longitudinal changes in average VARscores (**A** and **B**) and percentage peptide hits (**C** and **D**) for selected human (*n* = 18, 24; **A** and **C**) and macaque (*n* = 6; **B** and **D**) viral species were shown in heatmaps. Groups of MeV-infected macaques were not treated (WT MeV; *n* = 6), treated with RDV either prophylactically (d3–d14; RDV PEP; *n* = 5), or treated at the onset of disease (d11–d22; RDV LT; *n* = 3). Sham control animals (*n* = 4) served as uninfected, untreated controls. Human viral species were selected if the VARscore was over 2 or it PPH was over 10% at any time point in any macaque. The thresholds were chosen based on MeV VARscore and PPH values of pre- and post-MeV infection samples to select for the most important viral species for visualization. All macaque viral pathogens were selected for visualization. DPI, days postinfection; RM, rhesus macaque.

For each plasma sample, we derived the viral aggregate reactive score (VARscore), total peptide hits (TPH), and percentage peptide hits (PPH) per viral species from VirScan raw output files (Figure 1). VARscore is calculated as the comparison of mean log_2_ fold changes of virus-specific peptides versus random peptides in the VirScan library (18). TPH is defined as the total number of peptide tiles considered “hits” per viral species, while PPH is calculated as the percentage of TPH by the total number of peptide tiles per viral species. Essentially, the higher the VARscore, TPH or PPH, the more robust the plasma antibody (IgG) recognition to the viral species, proteins, or peptide tiles.

From 572 human viral species and strains, we identified 18 and 24 human viral species and strains with a VARscore > 2 and PPH > 10, respectively, at any time point in any macaque (Figure 1, A and C). Together with all of the 6 macaque viral species (Figure 1, B and D), longitudinal changes in average VARscore and PPH were visualized in Figure 1. Peptide hit per viral protein analysis of the 7 top viral species that overlapped in both VARscore and PPH datasets suggested that the high VARscore and PPH values of human respiratory syncytial virus (RSV) and simian foamy viruses (SFV) type 1 and 3 were likely results of prior infections (Supplemental Figure 1; supplemental material available online with this article; https://doi.org/10.1172/jci.insight.190740DS1).

Unexpectedly, there was antibody binding to a range of human viruses that are known to not infect RMs, such as human hepatitis B virus in the WT MeV and RDV PEP groups (Figure 1A). As a human-specific pathogen, hepatitis B is transmitted through blood or bodily fluid exchange (19) and, thus, unlikely that these animals were incidentally exposed. Therefore, these results were potentially due to background or cross-reactive antibody binding. Overall, multiple longitudinal changes in average VARscores and PPH were identified, with the sham control macaque samples providing a baseline with which to compare the samples from MeV-infected macaques.

### Effect of RDV prophylaxis and LT in MeV infection on plasma antibody to non-MeV pathogens.

**Figure 2:**
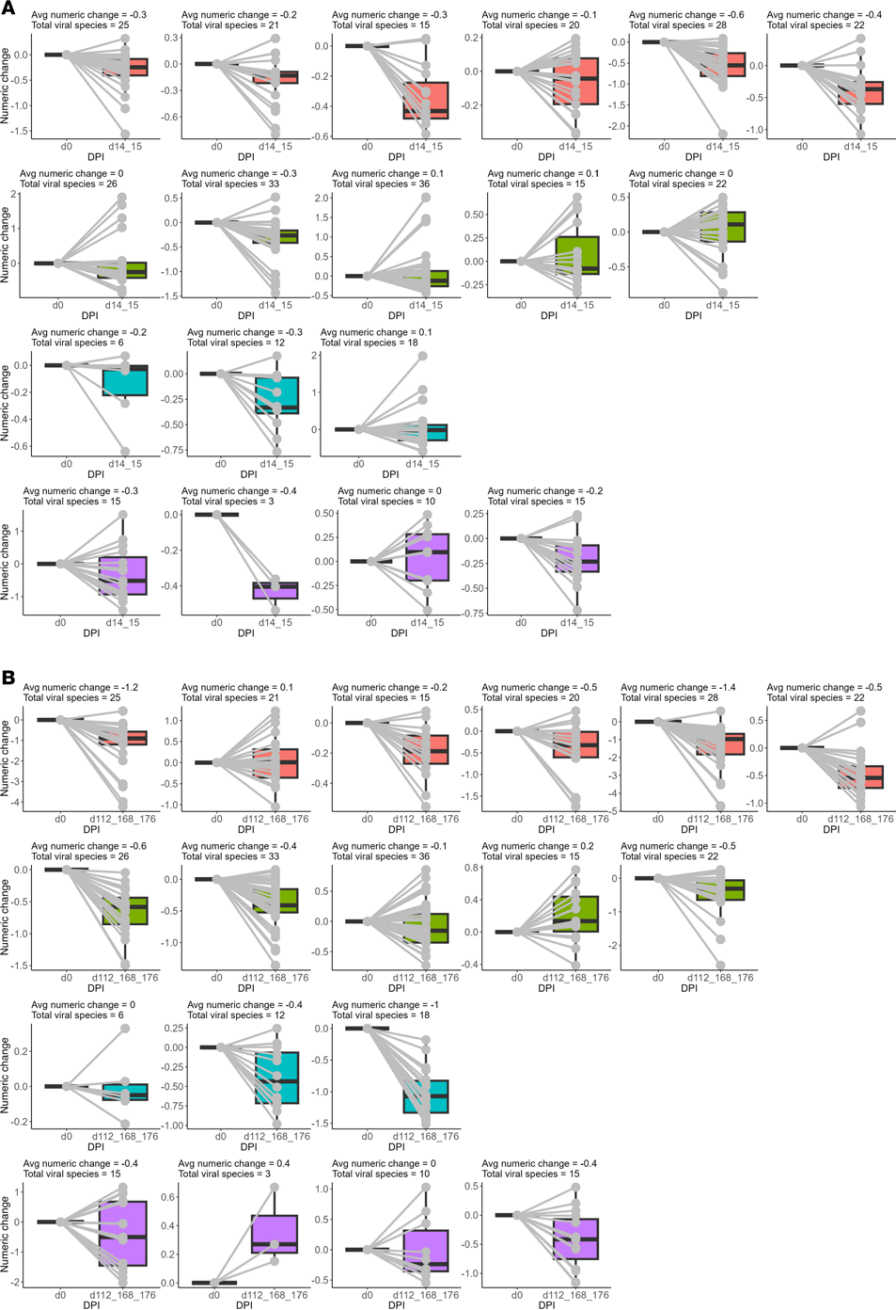
Numeric changes in VARscores from before infection to an early or late time point. (**A** and **B**) Box plots show average change in VARscores of each individual macaque for selected viral species (VARscore >1 at d0) to an early time point (d14/d15) (**A**) and the last time point (d112/d168/d176) (**B**). Data for each macaque was represented per box plot. All selected viral species were normalized and, thus, set to have a value of 0 at d0. Each viral species was represented by a gray line. Average numeric change per macaque and the total number of viral species that met the threshold requirement are indicated at the top of each box plot. Color in each box plot represents macaques in the WT MeV (pink), RDV PEP (green), RDV LT (blue), and Sham control (purple) groups. Avg, average; DPI, days postinfection.

**Figure 3:**
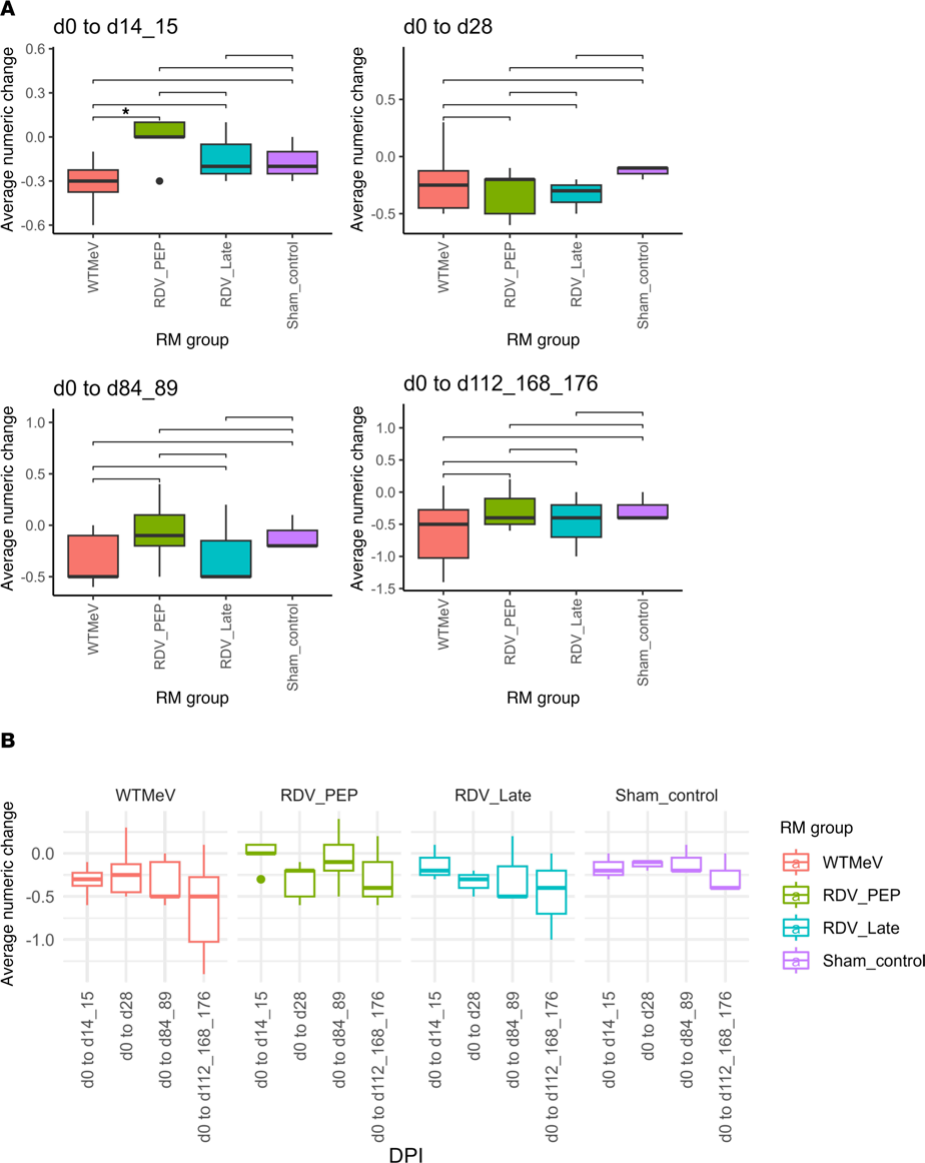
Longitudinal changes in VARscores from before infection to all time points. (**A** and **B**) Since d84/d89 sample from 1 RM was not available, the WT MeV group statistics of d0 to d84/d89 contained only 5 animals. Also, 1 RM from the Sham control group was omitted from this analysis, as less than 5 viral species had a VARscore of over 1 at d0. Statistical differences between time points were evaluated by Wilcoxon rank-sum test (**A**). Nonsignificant results were not shown; *P < 0.05. DPI, days postinfection.

To measure the longitudinal effects of WT MeV infection and RDV administration on the entire antibody repertoire to non-MeV pathogens, we measured the numeric changes in VARscores in the human library from preinfection to an early time point (2 weeks after infection at d14/d15) (Figure 2A) and to the last time point (15–25 weeks after infection) for each macaque (Figure 2B). Viral species were selected if VARscore was over 1 at day 0 for each macaque. Macaques with fewer than 5 selected viral species were excluded from subsequent analysis in Figure 3, A and B, as they had a limited number of viral species for comparison between 2 time points.

After WT MeV infection alone, animals experienced an average numeric change in VARscore among selected viral species of –0.32 (range: –0.6 to –0.1; early time point) from d0 to d14/15 and –0.62 (range: –1.4 to 0.1; late time point) from d0 to d112/168/176, a much greater decrease than the sham control group at –0.17 (range: –0.3 to 0; early time point) and –0.26 (range –0.4 to 0; late time point) (Figure 3, A and B). Average numeric change was significantly lower in the WT MeV than RDV PEP group from d0 to d14/15. Because longitudinal total IgG levels were stable (Supplemental Figure 2), this result demonstrated depletion of previously acquired antiviral immunity upon WT MeV infection, consistent with our previous study of immune amnesia to human pathogens induced by WT MeV infections in humans and macaques (14).

For PEP-treated MeV-infected macaques, the average numeric change in selected viral species VARscores was –0.02 (range: –0.3 to 0.1; early time point) and –0.28 (range: –0.6 to 0.2; late time point), approximately the same as for the sham control macaques and less than untreated MeV-infected macaques. For late-treated macaques, the average numeric change was –0.13 (range: –0.3 to 0.1; early time point) and –0.47 (range: –1 to 0; late time point), a result similar to untreated MeV-infected macaques. Therefore, RDV prophylaxis (early treatment) decreased the loss in amount of antibody to non-MeV pathogens after MeV infection. There was no indication that later RDV treatment had a similar effect.

### Antibody response to MeV.

**Figure 4:**
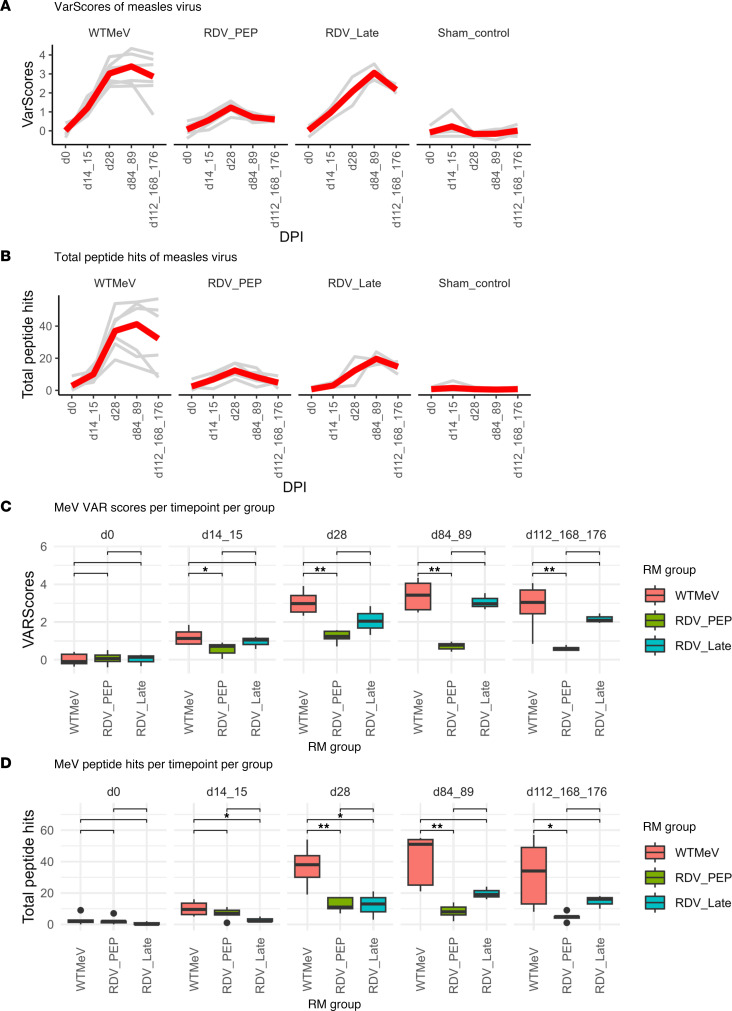
Longitudinal MeV-specific VARscores and average peptide hits per RM group. (**A**–**D**) Longitudinal MeV-specific VARscores (**A** and **C**) and average peptide hits (**B** and **D**) per macaque group. In **A** and **B**, gray lines represent values from each macaque, and the red line indicates the average values from all macaques in each group. Wilcoxon rank-sum test was employed to compare values between 2 groups (**C** and **D**). Nonsignificant results were not shown; *P < 0.05, **P < 0.01. DPI, days postinfection.

After examining the global antibody repertoire changes against a range of viral peptides, we examined the MeV-specific VARscores and TPHs (Figure 4). The greatest increase in MeV-specific antibodies, measured using the VARscore, was in the untreated WT MeV-infected macaques (Figure 4, A and B), followed by the RDV LT group and lastly the RDV PEP group, which had a significantly diminished antibody response when compared with the WT MeV macaques across longitudinal time points from d28. Uninfected control macaques showed no change over time and served as the longitudinal baseline/negative control. The trend was consistent for macaques within the same group.

MeV-specific TPHs were the greatest for WT MeV-infected macaques and were decreased in both PEP and late RDV treatment groups (Figure 4, C and D). Despite that, peak MeV-specific VARscore and TPHs for both WT MeV and RDV LT groups occurred at week 12, and the RDV PEP group occurred at week 4, indicating ongoing development of the antibody response long after clearance of infectious virus 2–3 weeks after infection for the former 2 groups (17). Therefore, the administration of RDV, especially early in the infection cycle (RDV PEP group), limited the humoral response against MeV.

### RDV infusion led to reduced magnitude and breadth of MeV-specific antibodies.

**Figure 5:**
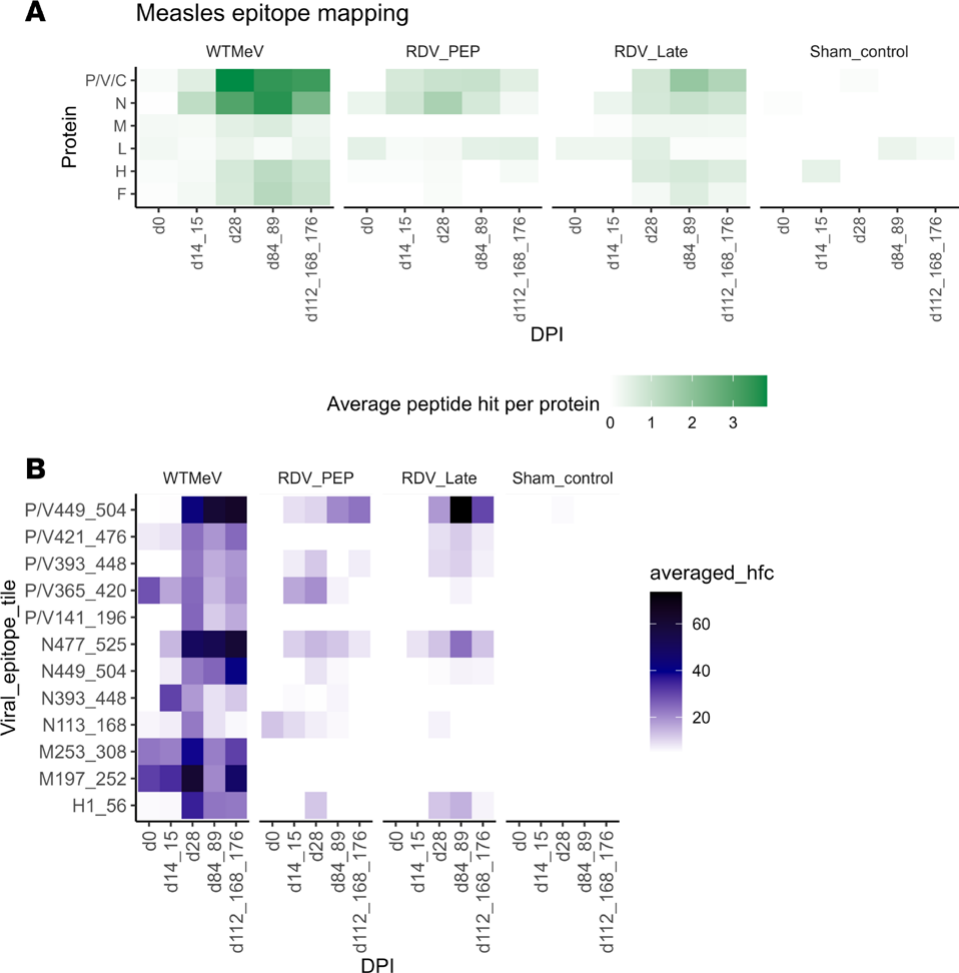
Longitudinal average peptide hits to all MeV proteins and selected viral peptides. (**A** and **B**) Longitudinal average peptide hits to all MeV proteins (**A**) and selected MeV viral peptides (**B**) per RM group, visualized by heatmaps. Of note, P encodes 2 nonstructural proteins; V and C, thus P/V/C and P/V, are referring to the same protein peptide tiles of P. In **B**, MeV viral peptides were selected for analysis when the average hit fold change was > 50 at any time point for any macaque. DPI, days postinfection; hfc, hit fold changes.

To further examine the effects of RDV administration on the development of MeV-specific antibodies, antibody reactivity to MeV viral proteins and peptides was analyzed (Figure 5). In all groups, the N protein was most immunogenic with detection of antibody 2 weeks after infection with the highest average peptide hit (Figure 5A). The next most immunogenic protein was P/V/C with reactivity detected by d28 in all groups except control. Antibodies reacting with H, M, and F proteins were less abundant in general. These findings are consistent with the antibody reactivity gradient that has been described for MeV previously (11). The MeV antibody profile of the RDV LT group resembled the WT MeV group profile but with less reactivity against MeV protein targets such as N and P/V/C. In the RDV PEP group, moderate antibody reactivity to N and P/V/C was detected, comparable with the RDV LT group. However, antibody reactivity to the M, H, and F proteins was mostly undetectable, suggesting that early administration of RDV limited both the magnitude and breadth of MeV-specific antibody responses.

To determine which linear MeV peptide tiles were most targeted by antibodies in the samples, we identified all peptide tiles with an average hit-fold change (HFC) value of over 50 at any time point in any macaque (Figure 5B). In the WT MeV group, binding antibodies were mostly targeting N477-525, P/V449-504, and H1-56 peptide tiles, consistent with previous observations in WT MeV-infected macaques (11). WT MeV-infected macaques also exhibited the greatest diversity of binding peptides, with high HFC values to peptide tiles P/V421-476, P/V393-448, P/V365-420, and N449-504. The RDV LT group macaques had a similar, but diminished, peptide binding profile to the WT MeV group, while the RDV PEP group had little detectable antibodies to H1-56.

### Potential MeV-neutralizing antibody targets in H and F proteins.

**Figure 6:**
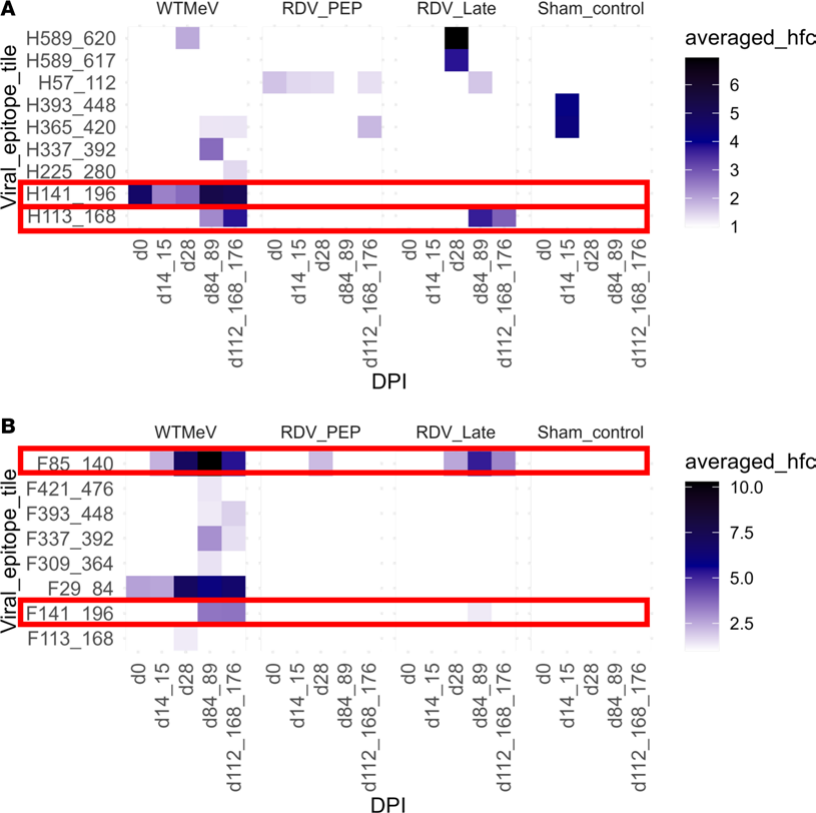
Longitudinal average peptide hits to selected MeV peptides in the H and F proteins per RM group. (**A** and **B**) Peptides for H protein (**A**) and F protein (**B**) with an average reactivity of HFC > 2 at any time point in any macaque were selected. The top 3 peptide tiles, N477-525, P/V449-504, and H1-56, were reported in Figure 5B and, thus, were excluded from the analysis. Top potential neutralizing targets were highlighted by red boxes. DPI, days postinfection; hfc, hit fold changes.

Because the H and F proteins are the targets for MeV-specific neutralizing antibodies (19), we identified H (Figure 6A) and F (Figure 6B) peptide tiles with antibody hits. Antibody reactivity broadened over time with reactivity to H1-56 strongly present at d28, while antibody to H113-168 appeared later at D84/89 for the WT MeV and RDV LT group, but not the RDV PEP group macaques. Antibody reactivity to F85-140appeared early in infection at d14/d15 for the WT MeV group and at D28 in the RDV LT group and peaked at d84/d89 for both groups; antibody reactivity to F29-84 was only found in WT MeV RMs. Also, as expected, fewer H and F peptide tiles were identified in the RDV PEP group.

## Discussion

MeV replicates in lymphoid tissue and has profound effects on the immune system, with loss of plasma antibodies to other pathogens, while generating long-term protective immunity to MeV reinfection. To assess the effect of early treatment and LT with RDV after infection on the loss of antibody to other pathogens as well as on the development of the antibody response to MeV, VirScan technology was expanded to include macaque pathogens and was used to analyze longitudinal responses of macaques infected with WT MeV and treated with the nucleoside analog RDV either prophylactically or at clinical disease onset. Longitudinal plasma antibody reactivity to a range of human and macaque viral peptides was examined in 4 groups of macaques (WT MeV, PEP, LT, and Sham control) and showed that early, but not late, treatment suppressed loss of previously developed non-MeV viral humoral immunity but also suppressed production of MeV-specific antibodies, likely due to the suppression of viral replication thus viral proteins available to stimulate the MeV-specific humoral response (17). Therefore, it suggests a potential role of early RDV treatment in deterring the development of immune amnesia and preservation of LLPCs, particularly in more clinically severe measles, which was demonstrated to have a more profound effect on antibody diversity than less severe measles (14).

The effect of RDV on MeV antibody production was distinct across treatment groups and for different MeV proteins. Peak MeV-specific antibody reactivity occurred at d84/d89 in both WT MeV and RDV LT groups, and d28 in the RDV PEP group, albeit with a lower peak in the latter. Also, while N-specific antibody was produced early, H-specific antibody appeared later, supporting the hypothesis that persistent MeV-specific RNA in PBMC and lymphoid tissues leads to production of viral protein and maturation of the long-lasting humoral responses (7, 15, 20). Furthermore, WT MeV induced the greatest breadth of antibodies across binding targets (polyclonal), while the RDV PEP group has the lowest breadth and mounted low levels of antibodies to M, H, or F. It is not known whether the reduced levels and diversity of MeV antibodies in the RDV PEP group are still sufficient to protect against MeV challenge, but levels of neutralizing antibody are within the range predicted to be protective (17).

Further analysis of the top 3 MeV peptide tiles showed that none is expressed on the virion surface. H1-56 is located within the intravirion and transmembrane region of the H protein (21), while N477-525 and P/V449-504 contain 2 known interacting protein sequences within the virion: N477-505, a sequence relatively conserved among the morbilliviruses, and P457-507, the extreme C-terminal domain, also known as the XD region (22, 23). Such observations preclude these peptide tiles as canonical neutralizing targets. Nonetheless, both N477-525 and H1-56 are known to contain MeV-specific T cell epitopes: 3 MeV-specific CD8 T cell epitopes, N493-512 (24), N512-526 (24), and H30-38 (25), and 1 vaccine-MeV-specific CD4 T cell epitope, H27-41 (26). It is therefore hypothesized that these peptide tiles are likely to be nonneutralizing antibody (NNAb) targets with potentially important Fc-mediated effector functions, including antibody-dependent cellular phagocytosis (ADCP) and intracellular neutralization. In ADCP, opsonized viral particles or debris is recognized by phagocytes or antigen-presenting cells (APC) such as macrophages, DCs, and B cells, leading to virolysis and/or antigen presentation to T cells (27). B cells acting as APC for validated MeV-specific T cell epitope peptides also supports the hypothesis/phenomenon of preferential association of adjacent T and B cell epitopes (28) and implies that there might be a way to predict the antibody repertoire from MHC1/2 T cell epitope interactions (or vice versa). In intracellular neutralization, 1 mechanism involves the binding of the intracellular NNAb-virus complex to TRIM21 (an E3 receptor ligase), leading to the proteasomal degradation of such NNAb-virus complex and the subsequent enhanced antigen presentation on MHCs (29). Specifically, nucleoprotein-specific NNAbs, commonly induced upon infection of enveloped viruses, have been demonstrated to confer protection via the TRIM21 pathway in many viral infections: lymphocytic choriomeningitis virus, adenoviruses, influenza virus, and paramyxoviruses (30, 31). Since both CD4 and CD8 T cells are instrumental in the maturation of MeV-specific immune responses (32), NNAbs are likely to play an important role in engaging with cellular immunity via ADCP, intracellular neutralization, or other mechanisms.

Our analysis identified potential neutralizing targets on H and F proteins, in addition to nonneutralizing targets. Notably, several neutralizing monoclonal antibodies (mAbs) have been validated to bind to MeV-specific epitopes within top peptide tiles H141-196, H113-168, F29-84, and F85-140 (Figure 6). For instance, mAbs mab48 and I-44 are known to react with the peptides H126-135 and H185-195, respectively (33, 34); mab77, which was extensively characterized via cryoelectron microscopy, has been shown to mediate neutralization by first forming contacts with amino acid residues such as W27, G37, and S40 on the F2 domain and L123 and V125 of the fusion peptide of the F protein via hydrogen bonds in the prefusion state, arresting the refolding process at an intermediate state, and preventing membrane fusion and viral entry (35). VirScan, thus, validated some epitope regions as MeV-specific neutralizing targets and has the potential to map binding and neutralizing epitopes for other viral diseases.

One limitation of this study is the focus on antibody detection using linear 56 amino acid peptide sequences, providing little room for protein folding beyond secondary structures and limiting detection of epitopes that involve discontinuous sequences and/or greater than 56 amino acid residues of each peptide tile; thus, the VirScan antibody data represent a fraction of the total antiviral antibody repertoire. However, the tradeoff of evaluating antibodies to larger folded proteins is the loss in single-epitope resolution and, thus, loss of detection of monoclonal antibody per peptide tile. Our current approach, therefore, allows for granular epitope mapping. The lack of further experimental validations such as ELISA and neutralization assays also limited the understanding of the seroneutralizing properties of the entire antibody repertoire at baseline and longitudinal time points; future work could include such characterization of antibody reactivity to individual viruses or viral proteins using established immunological assays.

Additionally, a practical limitation to the implementation of RDV PEP is the requirement for i.v. administration. Further investigation of antivirals that can be administered orally — such as the clinical-stage oral prodrug of the GS-441524 nucleoside obeldesivir, which metabolizes into the same active triphosphate species as RDV and demonstrated in vivo efficacy that mirrors RDV in nonclinical viral challenge models (36); the broadened-spectrum paramyxovirus polymerase inhibitor GHP-88309, which demonstrated efficacy in a canine-distemper virus-ferret model (37); or MeV RNA-dependent RNA-polymerase inhibitor ERDRP-0519 (38) — would be of potential interest. Lastly, while RDV resistance to MeV has not been assessed, RDV has demonstrated a high barrier to resistance in SARS-CoV-2 based on published sequence analyses from COVID-19 clinical studies (39–41); also, it is not anticipated to be of great concern in MeV due to its low mutation rate (42). More importantly, vaccination with the lab-adapted genotype A strain of MeV from 1954 (viral strain: Edmonston) has helped eradicate 20 of the 24 known MeV genotypes and continues to confer protection against all currently circulating genotypes (43). Altogether, it suggests that MeV viral escape to RDV remains a highly improbable event, though mutations in epitope regions of circulating viruses is shown to affect the effectiveness of vaccine-induced T cell immunity (26). Effect of RDV or any potential antivirals on MeV viral escape should therefore be continuously monitored.

In conclusion, this study highlights a potential role of early treatment to prevent long-term sequelae from MeV infection, specifically in immunocompromised individuals with persistent infections to facilitate viral clearance (17). Also, it emphasizes the public health importance of maintaining high measles vaccination coverage and developing effective antiviral treatments for MeV, a viral infection for which no antivirals are currently FDA-approved.

## Methods

### Animals, infection, and procedures

#### Sex as a biological variable.

Plasma samples from a total of 18 juvenile RMs (*Macaca mulatta*) were studied. Fourteen macaques (8 male, 6 female) from the Johns Hopkins University Primate Breeding Facility were previously infected intratracheally with the Bilthoven strain of WT MeV (1 × 10^4^ tissue culture infectious dose 50%; C2 genotype) and treated with RDV i.v. for 12 days either prophylactically (from d3 to d14 after infection; *n* = 5) at the time of disease onset (from d11 to d22 after infection; *n* = 3) or with vehicle (*n* = 6) (17). Four longitudinally followed control macaques were neither infected nor treated. Bilthoven (C2 genotype) was chosen because this genotype has historically been used in the laboratory to elucidate MeV immunopathology in a well-characterized macaque model. The findings in male and female animals were similar.

For all procedures, monkeys were sedated with 10–15 mg/kg ketamine or ketamine/dexmedetomidine (0.025 mg/kg) i.m. Heparinized blood was collected from the femoral vein before infection and every 3–14 days after infection for up to 6 months. Plasma and PBMCs were isolated by whole blood gradient centrifugation on Lympholyte-Mammal (Cedarlane Lab). Plasma was frozen at –20°C for subsequent analysis.

Of note, macaques were not vaccinated against any infectious diseases at the time when samples were collected. Furthermore, all monkeys were confirmed negative for anti-MeV antibody at baseline.

### Enzyme immunoassay

IgG in plasma was quantified using an enzyme immunoassay for RM IgG according to manufacturer’s instructions (Molecular Innovations, RSIGGKT).

### VirScan libraries

VirScan was expanded to include macaque pathogens and used to analyze antibody reactivity in plasma before and after WT MeV infection, with or without early or late administration of RDV. The VirScan PhIP-Seq methodology uses oligonucleotide library synthesis to generate proteomic-scale peptide libraries that can be expressed on T7 bacteriophage (44). These T7 bacteriophage peptide libraries are mixed with an antibody containing specimen (i.e., plasma) and antibodies bind to target epitopes expressed by the bacteriophage clones. Bacteriophages with bound antibodies are immobilized via immunoprecipitation using protein A/G beads that capture the Fc region of the antibodies, and immobilized bacteriophage are PCR-amplified and analyzed by high-throughput deep DNA-Seq to determine antibody-peptide interactions through peptide abundances, read counts, and fold-changes.

To develop the bacteriophage library, oligonucleotides, representing the virus peptide tiles corresponding to the original VirScan library and additional macaque pathogens, were synthesized by Agilent (human viruses) or Twist (macaque pathogens). Viral peptide tiles were provided as a pool, before being PCR amplified and ligated into T7 bacteriophage-display vector DNA, packaged into phage particles, and amplified in *E*. *coli* to form the VirScan phage library. The original 56mer VirScan human-pathogen library contains 115,753 peptides, and the macaque pathogen library contains 5,682 peptides.

### Macaque plasma screen and immunoprecipitation

Negative controls lacked antibody input (“mock IPs”) to obtain background binding information for each phage-displayed peptide. We used healthy human serum collected from the National Institute of Allergy and Infectious Diseases (NIAID) Vaccine Research Center (VRC) as a positive technical control. Macaque plasma samples were diluted 1:20 in PBS. Controls were randomly assigned to a position on a 96-well plate to reduce the potential for positional artifacts. The macaque and VirScan libraries were mixed with diluted plasma at 1 × 10^5^-fold library coverage. Approximately 20 billion pfu per well are combined with 2 μg IgG in diluted plasma samples and incubated overnight at 4°C. Antibody-bound phage were immunocaptured with protein A/G-coated magnetic beads (Dyna beads, Invitrogen), and any unbound nonimmobilized phage were washed away. A single-step PCR added index primer and barcodes for Illumina sequencing. Samples were then pooled and purified using a PCR clean-up kit. Purified PCR products were submitted to the core facility at Johns Hopkins School of Medicine for quantification and deep sequencing.

### Data analysis

Longitudinal plasma samples (d0, d14/d15, d28, d84/d89, and d112/d168/d176; except the 92F d84/d89 sample) from each group were subjected to experimental processes of VirScan as detailed above, with mock control reactions (beads only; negative controls). Several VirScan output files were generated for each 96-well immunoprecipitation plate, including raw read count files, fold change files, hits/hits fold change (HFC) files, and VARscores files.

Raw fastq files were first demultiplexed and aligned to generate a raw read counts file for each peptide in both the human VirScan and macaque libraries, whereby read counts for each peptide were determined via identical matching of the first 50 nucleotides of peptide coding DNA. Fold change files were then generated through the exact test for the negative binomial distribution with the R package, EdgeR (45). We have determined the standard definition of a “hit” (i.e., significant enrichment of this viral peptide by antibodies in the sample) as fold change in raw sequencing read counts compared with a plasma-free control of > 5, a total number of sequencing read counts > 15, and adjusted *P* value for enrichment of the peptide compared with a plasma free control < 0.001. VARscore is a measure of the overall level and breadth of antibody reactivity across the viral species (18, 46) and was generated through the comparison of mean log_2_ fold change values for virus-specific peptides to the distribution of expected mean log_2_ fold change values for randomly selected peptides in VirScan (https://github.com/wmorgen1/ARscore; commitID 982046d). For each viral species, the higher the VARscore, the more likely the viral species is significantly targeted by antibody responses.

With the raw fold change files, 3 additional datasets were generated: hits/HFC files, TPH, PPH files. HFC files are equivalent to fold change files, except that HFC values of peptides that are not considered “hits” are set to a fold change value of 1. Thus, for each viral peptide tile, a HFC value of over 1 was considered a “hit,” suggesting that antibodies significantly bind to and target this viral peptide tile. TPH is defined as the total number of peptide tiles considered as “hits” per viral species or per viral protein in each viral species in each sample, and average of TPH per macaque group is defined as average peptide hits. PPH was calculated through normalization of TPH by the total number of peptide tiles per viral species. For instance, if there were 30 peptide tile hits out of the 408 peptide tiles in MeV in 1 sample, TPH and PPH values would be 30 and 7.3%, respectively.

In total, antibody reactivity against 572 viral species and strains was examined (566 in the human and 6 in the macaque libraries; Supplemental Table 1). In the VARscore dataset, 349 viral species were selected for further examination, with top VARscore human viral species defined as viral species that achieved a VARscore > 2 at any time point in any macaque. Based on this empirical cut-off, there were 18 viral species considered as top human viral species. No cutoff was set for the macaque viral species, and all 6 viral species from the RM library were visualized. In the PPH dataset, 404 viral species were included for further examination, with the top PPH human viral species defined as viral species that had over 40 viral peptide tiles and PPH > 10% at any time point and in any macaque. There were 24 viral species considered as top human viral species in the PPH dataset. Of note, VirScan libraries contain viral peptide tiles from the same viral protein of different viral genotypes.

Lastly, in comparing antibody reactivity between the first and last time points per macaque, numeric change in VARscore was calculated per selected viral species, which were selected based on VARscore > 1 in d0 per macaque. Macaques with fewer than 5 viral species selected were not included for subsequent statistical analysis.

### Statistics

Preliminary bioinformatic analysis with statistical inference and graphical visualization to analyze the longitudinal VARscore per selected viral species, TPH per viral species or per viral protein, and HFC per viral peptide tile in each treatment group and macaque were conducted using R (Version 4.2.2; 2022) and R packages, such as dplyr, ggplot2, ggpubr, tidyverse, readr, stringr, gridExtra, and ggh4x. The Wilcoxon rank-sum test was used to compare values between 2 samples, and the Kruskal-Wallis test was used for global comparisons.

### Data availability

Supporting data values behind any reported means can be found in the Supporting Data Values file. All R codes, graphs, and datasets are currently published in https://github.com/Andy-ChanKP/VirScan_RDV_Analysis (commitID 16717b0).

### Study approval

All procedures were reviewed and approved by the Johns Hopkins University IACUC and conducted in accordance with guidelines in the Animal Welfare Regulations and the *Guide for the Care and Use of Laboratory Animals* (National Academies Press, 2011) within a fully Association for Assessment and Accreditation of Laboratory Animal Care–accredited (AAALAC-accredited) facility.

## Author contributions

DEG, HBL, DPP, and TC conceived of and participated in the design of the study. NAPA, JB, and SG collected and processed samples for the study. MT and LL performed the VirScan experiments. AKPC and LL performed data analysis. DEG and AKPC wrote the manuscript. WRM, MLB, KAMP, VCC, MSV, and MJM were consulted on data interpretation. All authors reviewed and edited the manuscript. Co–first authors agreed with the authorship orders to best reflect the effort put into the preparation of the manuscript.

## Supplementary Material

Supplemental data

Supporting data values

## Version 1 04/30/2025

In-Press Preview

## Version 2 06/09/2025

Electronic publication
