# Supplementary material for: Remdesivir postexposure prophylaxis limits measles-induced “immune amnesia” and measles antibody responses in macaques
Source: JCI Insight. 2025 Apr 22;10(11):e190740. doi: 10.1172/jci.insight.190740 (PMC12220960; doi:10.1172/jci.insight.190740)
Supplement: Supplemental data [file jciinsight-10-190740-s153.pdf]

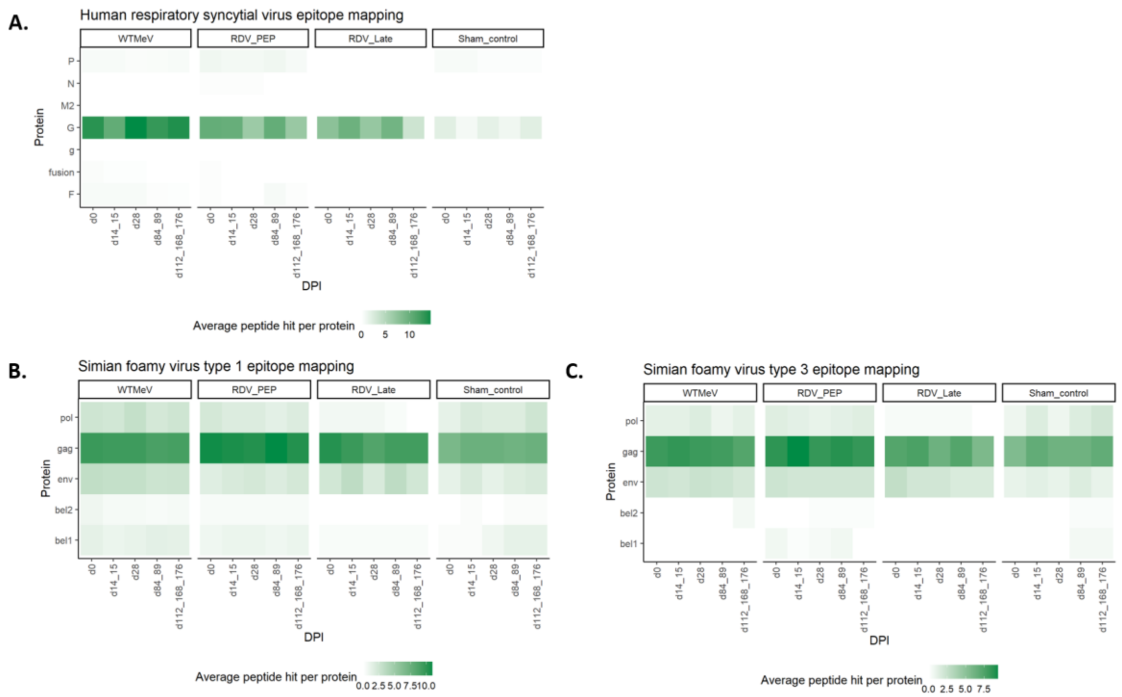

Supplemental Figure 1. Average number of viral peptide hits to (A) human respiratory syncytial virus, (B) simian foamy virus (SFV) type 1 and (C) SFV type 3 viral proteins per RM group longitudinally.

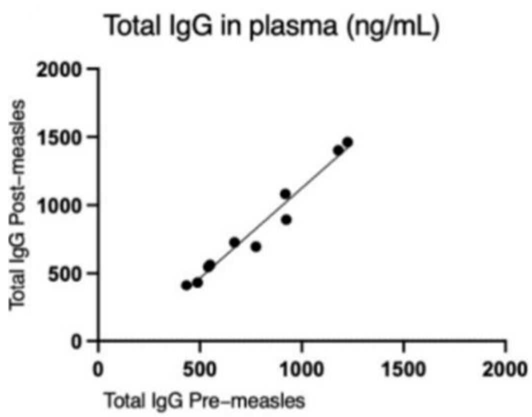

Supplemental Figure 2. Total macaque plasma IgG levels are unchanged after measles infection. Total plasma IgG were measured for 10 macaques on samples collected before (x-axis) and 112 d after (y-axis) WT MeV infection.

Supplemental Table 1. Number of peptide tiles per viral species

Taxon Species	Number of Peptide tiles
Adeno-associated virus	52
Adeno-associated virus - 3	22
Adeno-associated virus - 4	26
Adeno-associated virus 12	48
Adeno-associated virus 2	57
Adeno-associated virus 9	26
Adeno-associated virus VR-355	22
Aichi virus	138
Aichi virus 1	6
Aravan virus	23
Australian bat lyssavirus	138
Banna virus	206
Barmah forest virus	49
Bat coronavirus 1B	249
Bat coronavirus 279/2005	263
Bat coronavirus Rp3/2004	2
Berne virus	10
BK polyomavirus	98
Blue River virus	5
Bovine coronavirus	73
Bovine papular stomatitis virus	35
Bovine respiratory syncytial virus	13
Breda virus 1	8
BtVs-BetaCoV/SC2013	355
Bundibugyo ebolavirus	182
Bunyamwera virus	146
Bunyavirus La Crosse	164
Bunyavirus snowshoe hare	54
Camelpox virus	200
Canine adenovirus serotype 1	1
Canine kobuvirus US-PC0082	87
Cercopithecine alphaherpesvirus 2	124
Cercopithecine herpesvirus 1	1187
Cercopithecine herpesvirus 16	997
Cercopithecus erythrotis polyomavirus 1	2
Chandipura virus	127
Chapare mammarenavirus	118
Chiba virus	70
Chikungunya virus	907
Chimpanzee adenovirus Y25	129
Chimpanzee hepatitis B virus	18
Colorado tick fever virus	308
Convict Creek 107 virus	15
Coronavirus Neoromicia/PML-PHE1/RSA/2011	234
Cosavirus A	337
Cowpox virus	1268
Coxsackievirus A13	79
Coxsackievirus A16	156
Coxsackievirus A21	78
Coxsackievirus A24	79
Coxsackievirus A9	78
Coxsackievirus B1	76
Coxsackievirus B2	78
Coxsackievirus B3	125
Coxsackievirus B4	131
Coxsackievirus B5	78
Coxsackievirus B6	66
Crimean-Congo hemorrhagic fever virus	230
Dengue virus type 1	341
Dengue virus type 2	636
Dengue virus type 3	306
Dengue virus type 4	343
Dhori virus	74
Dugbe virus	226
Duvenhage virus	35
Eastern equine encephalitis virus	414
Ebola virus Yambio0402	4
Echo 9 virus	19
Echovirus 1	77
Echovirus 11	78
Echovirus 12	78
Echovirus 16	30
Echovirus 30	78
Echovirus 5	78
Echovirus 6	78
Echovirus 9	144
Echovirus E2	78
Ectromelia virus	35

Encephalomyocarditis virus	164
Enterovirus B	13
Epstein-Barr virus	2263
Equine coronavirus	254
Escherichia phage P1	12
European bat lyssavirus 1	75
Facey's Paddock virus	142
Feline adenovirus	20
Feline coronavirus	239
FinV707 virus	36
Four Corners hantavirus	35
Gammaherpesvirinae	1247
GB virus C	769
GB virus C variant troglodytes	105
GB virus Ccpz	41
Getah virus	132
Gibbon hepatitis B virus subtype ayw3q	34
Gorilla gorilla adenovirus B7	21
Gorilla gorilla gorilla polyomavirus 1	8
Gorilla hepatitis B virus	29
Gorilla rhadinovirus 1	5
Guanarito mammarenavirus	125
Hantaan virus	195
Hantavirus Monongahela-3	3
Hantavirus NFG357	15
HBV genotype H	8
Hendra virus	196
Hepatitis B virus	3761
Hepatitis B virus 146/Thailand	14
Hepatitis B virus ayw3	4
Hepatitis B virus genotype A1 subtype adw	37
Hepatitis B virus genotype A1 subtype adw2	32
Hepatitis B virus genotype A2	20
Hepatitis B virus genotype A2 subtype adw	12
Hepatitis B virus genotype A2 subtype adw2	80
Hepatitis B virus genotype A3	78
Hepatitis B virus genotype B/C subtype adw	40
Hepatitis B virus genotype B1	42

Hepatitis B virus genotype B1 subtype adw	35
Hepatitis B virus genotype B2	92
Hepatitis B virus genotype B2 subtype adw	37
Hepatitis B virus genotype C	47
Hepatitis B virus genotype C subtype ad	5
Hepatitis B virus genotype C subtype adr	90
Hepatitis B virus genotype C subtype ar	32
Hepatitis B virus genotype C subtype ayr	25
Hepatitis B virus genotype C subtype ayw	35
Hepatitis B virus genotype D	78
Hepatitis B virus genotype D subtype adw	38
Hepatitis B virus genotype D subtype ayw	68
Hepatitis B virus genotype E	77
Hepatitis B virus genotype E subtype ayw4	44
Hepatitis B virus genotype F1	34
Hepatitis B virus genotype F1 subtype adw4	5
Hepatitis B virus genotype F2	118
Hepatitis B virus genotype F2 subtype adw4q	46
Hepatitis B virus genotype G	49
Hepatitis B virus genotype G subtype adw2	6
Hepatitis B virus genotype H	5
Hepatitis B virus genotype H subtype adw4	68
Hepatitis B virus strain cpz	9
Hepatitis B virus subtype adr	52
Hepatitis B virus subtype adw	4
Hepatitis C virus	167
Hepatitis C virus genotype 1a	205
Hepatitis C virus genotype 1b	707
Hepatitis C virus genotype 1c	201
Hepatitis C virus genotype 2a	212
Hepatitis C virus genotype 2b	197
Hepatitis C virus genotype 2c	108
Hepatitis C virus genotype 2k	108

Hepatitis C virus genotype 3a	202
Hepatitis C virus genotype 3b	107
Hepatitis C virus genotype 3k	107
Hepatitis C virus genotype 4a	107
Hepatitis C virus genotype 5a	206
Hepatitis C virus genotype 6a	213
Hepatitis C virus genotype 6b	107
Hepatitis C virus genotype 6d	107
Hepatitis C virus genotype 6g	107
Hepatitis C virus genotype 6h	107
Hepatitis C virus genotype 6k	107
Hepatitis delta virus	429
Hepatitis delta virus genotype I	58
Hepatitis delta virus genotype II	15
Hepatitis delta virus genotype III	19
Hepatitis E virus	638
Hepatitis E virus genotype 1	244
Hepatitis E virus genotype 2	87
Hepatitis E virus genotype 3	105
Hepatitis E virus genotype 4	86
Hepatitis E virus rat/R63/DEU/2009	58
Herpes simplex virus type 2	33
Horsepox virus	139
Human adenovirus 11	6
Human adenovirus 14	50
Human adenovirus 19	23
Human adenovirus 21	43
Human adenovirus 22	90
Human adenovirus 29	16
Human adenovirus 30	8
Human adenovirus 34	46
Human adenovirus 36	18
Human adenovirus 48	45
Human adenovirus 49	30
Human adenovirus 54	27
Human adenovirus 55	84
Human adenovirus 56	5
Human adenovirus 58	37
Human adenovirus 62	27
Human adenovirus 64	25
Human adenovirus 65	18
Human adenovirus 7a	4
Human adenovirus 8E	8

Human adenovirus A serotype 12	367
Human adenovirus A serotype 18	271
Human adenovirus A serotype 31	244
Human adenovirus B serotype 11	21
Human adenovirus B serotype 16	119
Human adenovirus B serotype 3	181
Human adenovirus B serotype 35	58
Human adenovirus B serotype 7	312
Human adenovirus C serotype 1	96
Human adenovirus C serotype 2	338
Human adenovirus C serotype 5	212
Human adenovirus C serotype 6	47
Human adenovirus D serotype 15	73
Human adenovirus D serotype 15/H9	11
Human adenovirus D serotype 17	64
Human adenovirus D serotype 8	183
Human adenovirus D serotype 9	83
Human adenovirus D37	79
Human adenovirus E serotype 4	362
Human adenovirus F serotype 40	353
Human adenovirus F serotype 41	355
Human astrovirus-1	192
Human astrovirus-2	39
Human astrovirus-3	37
Human astrovirus-4	44
Human astrovirus-5	40
Human astrovirus-6	62
Human astrovirus-7	32
Human astrovirus-8	88
Human astrovirus 1 Beijing/46/2005/CHN	5
Human astrovirus 1d	6
Human betaherpesvirus 6A	13
Human betaherpesvirus 7	178
Human calicivirus NLV/Bad Berleburg/477/01	5
Human coronavirus 229E	356
Human coronavirus EMC	500
Human coronavirus HKU1	1471
Human coronavirus NL63	351
Human coronavirus OC43	320
Human cosavirus A	81
Human cosavirus A11	10

Human cosavirus A12	10
Human cosavirus A13	10
Human cosavirus A14	10
Human cosavirus A15	20
Human cosavirus A16	10
Human cosavirus A17	10
Human cosavirus A18	10
Human cosavirus A19	76
Human cosavirus A20	75
Human cosavirus A21	10
Human cosavirus A22	10
Human cosavirus A23	10
Human cosavirus A24	10
Human cosavirus A3	10
Human cosavirus A5	10
Human cosavirus A6	10
Human cosavirus A7	10
Human cosavirus A8	10
Human cosavirus A9	10
Human cosavirus RdRp-1892	3
Human cytomegalovirus	4018
Human enterovirus 70	74
Human enterovirus 71	130
Human erythrovirus V9	10
Human hepatitis A virus genotype IA	184
Human hepatitis A virus genotype IB	47
Human hepatitis A virus genotype IIA	47
Human hepatitis A virus genotype IIB	70
Human hepatitis A virus genotype IIIA	66
Human hepatitis A virus genotype IIIB	60
Human herpesvirus 1	1825
Human herpesvirus 2	1458
Human herpesvirus 2 strain SN03	4
Human herpesvirus 3	376
Human herpesvirus 6A	1415
Human herpesvirus 6B	722
Human herpesvirus 7	1243
Human herpesvirus 8	1721

Human herpesvirus 8 type M	146
Human herpesvirus 8 type P	453
Human immunodeficiency virus type 1 group M subtype A	204
Human immunodeficiency virus type 1 group M subtype B	826
Human immunodeficiency virus type 1 group M subtype C	193
Human immunodeficiency virus type 1 group M subtype D	225
Human immunodeficiency virus type 1 group M subtype F1	162
Human immunodeficiency virus type 1 group M subtype F2	114
Human immunodeficiency virus type 1 group M subtype G	143
Human immunodeficiency virus type 1 group M subtype H	139
Human immunodeficiency virus type 1 group M subtype J	81
Human immunodeficiency virus type 1 group M subtype K	140
Human immunodeficiency virus type 1 group M subtype U	22
Human immunodeficiency virus type 1 group N	87
Human immunodeficiency virus type 1 group O	164
Human immunodeficiency virus type 2 subtype A	656
Human immunodeficiency virus type 2 subtype B	239
Human klassevirus 1	38
Human mastadenovirus B	12
Human mastadenovirus C	52
Human mastadenovirus D	48
Human mastadenovirus E	65
Human mastadenovirus F	19
Human metapneumovirus	144
Human papillomavirus type 1	82
Human papillomavirus type 10	78
Human papillomavirus type 11	98
Human papillomavirus type 12	61
Human papillomavirus type 13	96
Human papillomavirus type 14	63
Human papillomavirus type 15	79
Human papillomavirus type 16	199

Human papillomavirus type 17	81
Human papillomavirus type 18	108
Human papillomavirus type 19	83
Human papillomavirus type 20	51
Human papillomavirus type 21	60
Human papillomavirus type 22	86
Human papillomavirus type 23	88
Human papillomavirus type 24	92
Human papillomavirus type 25	68
Human papillomavirus type 26	76
Human papillomavirus type 27	64
Human papillomavirus type 28	80
Human papillomavirus type 29	80
Human papillomavirus type 2a	36
Human papillomavirus type 3	79
Human papillomavirus type 30	88
Human papillomavirus type 31	131
Human papillomavirus type 32	89
Human papillomavirus type 33	79
Human papillomavirus type 34	87
Human papillomavirus type 35	107
Human papillomavirus type 36	76
Human papillomavirus type 37	71
Human papillomavirus type 38	80
Human papillomavirus type 38b	6
Human papillomavirus type 39	104
Human papillomavirus type 4	86
Human papillomavirus type 40	68
Human papillomavirus type 41	92
Human papillomavirus type 42	114
Human papillomavirus type 43	90
Human papillomavirus type 44	58
Human papillomavirus type 45	94
Human papillomavirus type 47	83
Human papillomavirus type 48	82
Human papillomavirus type 49	82
Human papillomavirus type 5	84
Human papillomavirus type 50	84
Human papillomavirus type 51	88
Human papillomavirus type 52	110
Human papillomavirus type 53	104
Human papillomavirus type 54	86
Human papillomavirus type 55	35

Human papillomavirus type 56	95
Human papillomavirus type 57	59
Human papillomavirus type 58	145
Human papillomavirus type 59	115
Human papillomavirus type 5b	34
Human papillomavirus type 60	86
Human papillomavirus type 61	81
Human papillomavirus type 62	84
Human papillomavirus type 63	84
Human papillomavirus type 65	60
Human papillomavirus type 66	81
Human papillomavirus type 67	82
Human papillomavirus type 68	84
Human papillomavirus type 69	83
Human papillomavirus type 6a	6
Human papillomavirus type 6b	62
Human papillomavirus type 6c	4
Human papillomavirus type 7	78
Human papillomavirus type 70	89
Human papillomavirus type 72	82
Human papillomavirus type 73	84
Human papillomavirus type 8	90
Human papillomavirus type 81	5
Human papillomavirus type 82	91
Human papillomavirus type 9	82
Human papillomavirus type 94	28
Human papillomavirus type RTRX7	73
Human parainfluenza 1 virus	118
Human parainfluenza 2 virus	200
Human parainfluenza 3 virus	228
Human parainfluenza 4a virus	74
Human parainfluenza 4b virus	22
Human parechovirus 1	77
Human parechovirus 2	155
Human parvovirus B19	206
Human picobirnavirus	413
Human respiratory syncytial virus	673
Human respiratory syncytial virus A	143
Human respiratory syncytial virus B	139
Human rhinovirus 14	77
Human rhinovirus 16	76
Human rhinovirus 1A	29
Human rhinovirus 1B	77

Human rhinovirus 2	76
Human rhinovirus 23	127
Human rhinovirus 3	30
Human rhinovirus 89	67
Human rhinovirus A serotype 89	77
Human rhinovirus A39	77
Human rhinovirus B70	78
Human rotavirus A	28
Human SARS coronavirus	85
Human spumaretrovirus	103
Human T-cell leukemia virus 1	245
Human T-cell leukemia virus 2	201
Human T-cell leukemia virus 3	131
Human torovirus	43
Influenza A virus	5685
Influenza B virus	963
Influenza C virus	886
Isfahan virus	125
Jabora virus	7
Japanese encephalitis virus	219
JC polyomavirus	113
Junin mammarenavirus	134
KI polyomavirus	55
Kunjin virus	122
Kupe virus	144
Kyasanur forest disease virus	121
Lagos bat virus	227
Lake Victoria marburgvirus	562
Langat virus	122
Lassa mammarenavirus	633
Lassa virus	294
Lloviu cuevavirus	25
Lordsdale virus	88
Louping ill virus	149
Lymphocytic choriomeningitis virus	142
Macaca_mulatta_rhadinovirus	3
Macacine gammaherpesvirus 5	1196
Machupo virus	139
Madariaga virus	66
Madre de Dios virus	50
Maguari virus	3
Mamastrovirus 1	33
Mayaro virus	130

Measles virus	410
Mengo encephalomyocarditis virus	96
Merkel cell polyomavirus	216
Middle East respiratory syndrome-related coronavirus	1317
Mokola virus	7
Molluscum contagiosum virus	89
Molluscum contagiosum virus subtype 1	1987
Molluscum contagiosum virus subtype 2	17
Moloney murine leukemia virus	62
Monkeypox virus	290
Mumps rubulavirus	6
Mumps virus	228
Murray valley encephalitis virus	122
Necocli virus	5
New York virus	80
Nipah virus	199
Non-human primate Adeno-associated virus	78
Norovirus Hu/GI.4/1643/2008/US	7
Norovirus Hu/GII.4/HK/CU09N8/2009/CHN	3
Norovirus Hu/GII/2005/6894/Chelyabinsk/RUS	4
Norovirus Hu/GII/Moscow/7844/2005/RUS	4
Norovirus Hu/Saga/000313/2000/JP/2876	19
Norovirus MD145	60
Norwalk-like virus	67
Norwalk-like virus NLV/Honolulu/219/1992/US	19
Norwalk virus	88
O'nyong-nyong virus	260
Orangutan hepatitis B virus	42
Orf virus	2183
Orf virus OV/mi-90	12
Oropouche virus	243
Pan troglodytes troglodytes foamy virus	5
Panine betaherpesvirus 2	48
Parainfluenza virus 5	213
Phocid gammaherpesvirus	46

Pichinde mammarenavirus	118
Poliovirus type 1	574
Poliovirus type 2	156
Poliovirus type 3	139
Porcine adenovirus 1	1
Porcine adenovirus A serotype 3	7
Porcine hemagglutinating encephalomyelitis virus	16
Porcine picobirnavirus 1	3
Porcine picobirnavirus strain D4	1
Porcine picobirnavirus strain E4	1
Pseudocowpox virus	545
Punta toro phlebovirus	78
Puumala virus	302
Rabbitpox virus	50
Rabies lyssavirus	18
Rabies virus	500
Respiratory syncytial virus	7
Reston ebolavirus	470
Rhesus monkey rhadinovirus H26-100	8
Rhesus monkey rhadinovirus H26-101	4
Rhesus monkey rhadinovirus H26-95	4
Rhesus monkey rhadinovirus H26-96	2
Rhesus monkey rhadinovirus H26-97	2
Rhesus monkey rhadinovirus H26-98	2
Rhesus monkey rhadinovirus H26-99	2
Rhinovirus A	77
Rhinovirus B	77
Rift valley fever virus	228
Rosavirus A2	91
Rosavirus M-7	88
Ross river virus	185
Rotavirus A	1785
Rotavirus B	397
Rotavirus C	199
Rotavirus G1	4
Rotavirus X	205
Rubella virus	439

Sabia mammarenavirus	118
Sagiyama virus	91
Saimiri sciureus rhadinovirus 1	1
Saimiriine herpesvirus 1	6
Saimiriine herpesvirus 2	1406
Salivirus A	111
Salivirus csh-1	4
Salivirus FHB	84
Salivirus NG-F1	74
Sandfly fever sicilian virus	55
Sandfly fever Turkey virus	121
Sapovirus	85
Sapovirus Hu/Ehime/2K-814/2000	5
Sapovirus Hu/GII/Tokyo/08-762/2008/JPN	5
Sapporo virus	263
Semliki forest virus	134
Seoul virus	131
Simian adenovirus 24	8
Simian adenovirus 25	13
Simian adenovirus B	372
Simian adenovirus E22	12
Simian foamy virus	40
Simian foamy virus type 1	149
Simian foamy virus type 3	120
Simian hepatitis A virus genotype V	79
Simian mastadenovirus G	362
Simian T-cell lymphotropic virus 6	86
Simian T-lymphotropic virus 3	8
Simian virus 40	132
Sin Nombre orthohantavirus	84
Sindbis virus	147
Sindbis virus subtype Ockelbo	133
Southampton virus	89
St. louis encephalitis virus	121
Sudan ebolavirus	362
Swine hepatitis E virus	4
Tai Forest ebolavirus	226
Tanapox virus	937
Taterapox virus	9
Tick-borne powassan virus	121
Torque teno midi virus 1	33
Torque teno mini virus 1	30

Torque teno virus	216
Torque teno virus 1	27
Toscana virus	177
Una virus	5
uncultured picobirnavirus	16
Utinga virus	50
Utive virus	88
Uukuniemi virus	212
Vaccinia virus	1943
Vaccinia virus GLV-1h68	4
Varicella-zoster virus	1277
Variola virus	674
Venezuelan equine encephalitis virus	437
Vesicular stomatitis Indiana virus	254

Vesicular stomatitis New Jersey virus	127
West Nile virus	209
Western equine encephalitis virus	139
WU polyomavirus	89
Wyeomyia orthobunyavirus	8
Xenotropic MuLV-related virus	108
Yaba-like disease virus	451
Yaba monkey tumor virus	1111
Yellow fever virus	675
Zaire ebolavirus	501
Zaliv Terpenia virus	9
Zika virus	210
